# The future of scientific publication is Open Access, but needs diversity, equability and equality!

**DOI:** 10.1515/iss-2021-2038

**Published:** 2021-04-07

**Authors:** Joachim Jähne

**Affiliations:** Clinic for General and Digestive Surgery, Diakovere Friederikenstift and Henriettenstift, Marienstr, 72-90, D-30171Hannover, Germany

Almost 30 years ago, the physicist Paul Ginsparg, at the age of 36 years, installed a server at the Los Alamos National Laboratory to allow the free access to preprints in physics. That can be best described as the foundation of the Open Access movement. It was, however, the increasing financial burden of the “old” subscription model for universities, research institutions and libraries with annual price increases on the one and decreasing financial support on the other hand, which has been a strong argument for Open Access scientific communication, in particular, since the established publishers realized profit margins between 20 and 30% [1]. Subsequently, the Budapest Open Access Initiative in 2001 and the Bethesda Statement as well as the Berlin Declaration, both in 2003, argued for Open Access to scientific knowledge. In recent years, more and more scientific and research organizations as well as libraries from all over the world signed, for example, the Berlin Declaration—667 signatories as of January 2nd, 2021 [2].

Based on these developments, Open Access publications showed a rapid growth between 1993 and 2009, which, since the year 2000, reached an annual average increase of 18% for the number of journals and of 30% for the number of articles. Additionally, in 2009, Open Access articles accounted for almost 8% of all peer-reviewed journal articles [3]. It was calculated that increased Open Access publication results in substantial benefits to research findings [4]. The constant rise of Open Access publications turned out to be an undisputable fact. Between 2009 and 2018, the percentage of Open Access publications in total publications in many nations of the European Union as well as in the USA was as high as 40%, in some countries even close to 50%, and in clinical medicine, 44% of all articles are published Open Access [5]. By looking to these numbers, it becomes clear, that the future of publication in science, technology and mathematics as well as in medicine and in particular in surgery will be Open Access. Even Annals of Surgery, the most prestigious surgical journal worldwide, has recognized the importance of Open Access and launched an Open Access version in September 2020 [6]. It can be postulated, that Open Access increases visibility. Visibility creates citation, citation creates usage und usage creates impact factor [7]. With this in mind, we have to do our utmost to ensure high research quality to impact the right people. Open Access on its own does not do that, but quality research does. These features all together create knowledge which becomes a common good. However, since many questions are still not answered, it is up to us to shape the conditions for Open Access publications.

Just recently, some interesting proposals for the future of scientific publishing in the life sciences were suggested, and a transparent review-process, among others, was one suggestion to overcome outdated publishing processes [8]. Since the launch of “Innovative Surgical Sciences” in 2016, a transparent double-blind peer review process and publication of the reviews together with the article have been a major and among comparable surgical journals still unique feature of the journal. Additionally, the discussion on transparency should address the question of publishing preprints that are peer-reviewed by the scientific community in order to improve the manuscript as well as research activities. In the Covid-19 pandemic, for example, preprints have been essential for rapid dissemination of new findings. However, transparency in the review process is just one issue of relevance. Other factors of future publication refer to the significance of impact factors to assess scientific achievements and the question of negative results, which are often not published, but may be of importance for future research activities [9]. It becomes more and more evident, that perhaps downloads and other manuscript-oriented altmetrics are of greater interest than just impact factors. And even funding agencies like EMBO (European Molecular Biology Organization) value personal statements and motivation far more than publication history as they stated in a Lindau Nobel laureate discussion [9]. One other, and probably even more relevant issue refers to the participation in open research and Open Access. Up till now, research and publication are primarily US-, European- and Asian-centric. There are concerns that low and middle income countries despite the EIFL-initiative (Electronic Information for Libraries; https://www.eifl.net/) may be lost on the way to the future of publications simply because they cannot afford it. This relates in particular to publishing in a Gold Open Access/Article Processing Charges model. Therefore, there is a need to define opportunities for participation in order to make every participant visible [10], [11]. It can well be assumed that the scientific community will agree that Open Access is undoubtedly associated with full participation of everyone. Keeping this in mind we also have to acknowledge that Open Access for all cannot be established without financial efforts. At the end, “someone must pay for the costs of publishing all this ever-increasing research” [12]. The European Union (https://open-research-europe.ec.europa.eu/) advocates Open Access research and a rapid and transparent publishing process powered by the F1000research-initiative (https://f1000research.com/). Another important achievement is the PlanS or cOAlitionS concept (https://www.coalition-s.org/) which was, at least in 2019, almost unknown among the 50 Editorial Board members of the British Journal of Surgery [12]. Besides the fact that this is just one example for the old world of publication, PlanS poses in particular for surgery a major problem: only studies that are funded by public grants will have a chance for Gold Open Access publication, and the costs are covered by the grants. The same holds true for studies funded by the European Union, although the European Research Council just recently withdrew from PlanS (https://erc.europa.eu/news/erc-scientific-council-calls-open-access-plans-respect-researchers-needs). Grants from the European Union are still to be published Open Access, and the Article Processing Charges are eligible costs within the grants. Many surgical research papers from all over the world, however, cover clinical studies that are most often not funded at all. These papers would never have a chance to be published Open Access. The project DEAL (https://www.projekt-deal.de/about-deal/) tries to overcome these challenges by financing the publications of more than 900 publicly and privately funded academic research and governmental institutions. Up till now, the contract was signed by the publishers Wiley and Springer Nature with other publishers most likely to follow. Again, hospitals which are not affiliated to a university are not covered and, even more worrisome, the contract involves just the big publishers. That appears not to be fair, since Open Access argues for diversity, equability and equality. In a globalized world, in which digitalization is one of the most important innovations during the last years, everybody should have the same chances of participation irrespective of race, color, gender, ethnicity, cultural background, sexual orientation or financial possibilities. We at Innovative Surgical Sciences—Executive and Associate Editors as well as the staff of the publisher DeGruyter—consider these conditions as an obligation to shape the future of the journal as well as of the Open Access movement.
